# Altered expression levels of autophagy-associated proteins during exercise preconditioning indicate the involvement of autophagy in cardioprotection against exercise-induced myocardial injury

**DOI:** 10.1186/s12576-020-00738-1

**Published:** 2020-02-17

**Authors:** Jian-Qi Yuan, Yang Yuan, Shan-Shan Pan, Ke Cai

**Affiliations:** School of Kinesiology, Shanghai University of Sport, 399 Changhai Road, Shanghai, 200438 China

**Keywords:** Exercise preconditioning, Ischemia–hypoxia, Autophagy, Cardioprotection

## Abstract

Exercise has been reported to induce autophagy. We hypothesized that exercise preconditioning (EP)-related autophagy in cardiomyocytes could be attributed to intermittent ischemia–hypoxia, allowing the heart to be protected for subsequent high-intensity exercise (HE). We applied approaches, chromotrope-2R brilliant green (C-2R BG) staining and plasma cTnI levels measuring, to characterize two periods of cardioprotection after EP: early EP (EEP) and late EP (LEP). Further addressing the relationship between ischemia–hypoxia and autophagy, key proteins, Beclin1, LC3, Cathepsin D, and p62, were determined by immunohistochemical staining, western blotting, and by their adjacent slices with C-2R BG. Results indicated that exercise-induced ischemia–hypoxia is a key factor in Beclin1-dependent autophagy. High-intensity exercise was associated with the impairment of autophagy due to high levels of LC3II and unchanged levels of p62, intermittent ischemia–hypoxia by EP itself plays a key role in autophagy, which resulted in more favorable cellular effects during EEP-cardioprotection compared to LEP.

## Background

Strategies that encourage endogenous cardiac adaptations have increasingly been used as non-pharmacological therapies to mitigate the risks of cardiovascular events. One example, known as cardiac preconditioning, involves vigorous exercise prior to ischemic events to prevent lethal myocardial injury [1–4]. Exercise preconditioning (EP), which consists of both short-term and long-term repeated intermittent exercise, can reduce myocardial injury caused by exhaustive, continuous high-intensity exercise [1]. High-intensity exercise can risk myocardial health, as it inevitably results in an imbalance between increased oxygen consumption and insufficient oxygen uptake [5]. Single bouts of short-term, intermittent exercise may improve myocardial adaptation and tolerance and protect against the risks involved in exercise, such as the excessive ischemia–hypoxia caused by continuous high-intensity exercise [6].

Ischemic preconditioning (IP), also known as intermittent ischemia/reperfusion (I/R), can result in cardioprotection in the myocardium, alleviating subsequent myocardial ischemia–hypoxia-like injury or ischemia-induced infarction [7]. EP has been shown to induce an adaptive promotion that generates dual-protective windows similar to those generated during IP [8–10]. This EP-induced protection is associated with early exercise preconditioning (EEP), which occurs immediately after EP, and late exercise preconditioning (LEP), which occurs 12–24 h after EP [11]. Multiple mechanisms underlying the cardioprotective effect of EP have been described. Recently, mitophagy and Hsp70-assisted autophagy, processes known to be sensitive to ischemia–hypoxia stress, have been implicated in EP-induced cardioprotection [6, 12]. However, there is not yet clear evidence of the relationship between exercise-induced ischemia–hypoxia and autophagy.

Autophagy is an important cellular process that maintains energy homeostasis as an autologous protection mechanism. Studies have shown that autophagy is induced in ischemia–hypoxia heart tissue by various stress conditions [13]. Autophagy is a dynamic, time-sensitive process of autophagosome formation and engulfment and lysosomal fusion called autophagic flux [14]. There are four main ways to determine autophagy levels. The first is to measure the levels of Beclin1, a BH-3-only domain protein essential for autophagic induction. Beclin1 induces the engulfment of autophagic substrates by the primary phagophore [15]. The second is to monitor the conversion of microtubule-associated protein 1 light chain 3 (LC3) I to LC3II. The autophagosome is generated by the maturation of double-bilayers. The third way is to measure the amount of p62, the levels of which are inversely correlated with autophagic activity [16–18]. The final way is to monitor the levels of Cathepsin D, a representative proteinase abundantly expressed in myocardium that reportedly increases IP-cardioprotection [19].

While normal autophagy plays a key role in utilizing discarded products, abnormal autophagy aggravates cellular injury [20]. To determine the autophagic status of cardiomyocytes undergoing ischemia–hypoxia, the chromotrope-2R brilliant green (C-2R BG) staining was used in the myocardium, which could specifically detect ischemia–hypoxia cardiomyocytes, and four critical autophagy proteins (Beclin1, LC3, Cathepsin D, and p62) were measured by immunohistochemical staining and western blotting in the myocardium. Since exercise is known to induce autophagy, we hypothesized that the expression of autophagy-associated proteins may differ between EP and high-intensity exercise and result in distinct autophagic phenotypes due to different characteristics of ischemia–hypoxia.

## Methods

### Animals and grouping

All animal studies conformed with the Guide for the Care and Use of Laboratory Animals, published by the US National Institutes of Health (NIH Publication, 8th Edition, 2011) and were approved by the Ethics Committee for the Science Research at Shanghai University of Sport. Healthy 8-week-old male Sprague-Dawley rats (*n* = 150, Shanghai Sippr-BK laboratory animal Co. Ltd, China) weighing about 180 ± 13 g were housed at five rats per cage and kept at a constant temperature (22–24 °C) and humidity (40–70%) on a 12-h light/dark cycle.

### Experimental protocol

All rats were made to perform adaptive treadmill running (10 min at 15 m/min, 0% grade) for 5 days, followed by a day of rest. They were randomly distributed into six groups (*n* = 25) according to their body weights, and all of them underwent the procedures described below. All rats running began with a 5-min “warm-up” and ended with a 5-min “cool down” at 15 m/min on treadmills with 0% grade. All the rats were anesthetized by intraperitoneal injection of 10% trichloroacetaldehyde hydrate at a dose of 400 mg/kg and killed 0.5 h after they finally got off treadmill, except LEP group was killed 24 h later.

Group C (Control group): rats were placed on the stationary treadmill.

Group EEP (early exercise preconditioning): rats were made to run at 30 m/min on treadmills with 0% grade for four 10-min periods, which was 75% VO_2_max following previous description [6].

Group LEP (late exercise preconditioning): rats were made to run just as those in the EEP group were, then rest for 24 h.

Group HE (high-intensity exercise): rats were made to run last long. In the beginning, the treadmill speed was from 15 to 35 m/min within 5 min, and was kept at 35 m/min on treadmills with 0% grade for 3 h to produce myocardial injury of HE. The treadmill running at 35 m/min corresponded with approximately 80% VO_2_max in rats, that exercise located in the high-intensity as description [21].

Group EEP + HE (early exercise preconditioning plus high-intensity exercise): rats were made to run just as those in the EEP group were. Thirty minutes later, they underwent above described HE. This group was used to assess the cardioprotection of EEP against HE.

Group LEP + HE (late exercise preconditioning plus high-intensity exercise): rats were made to run just as those in the LEP group were. Twenty-four hours later, they underwent the above described HE. This group was used to assess the cardioprotection of LEP against HE.

After anesthetized, the abdominal cavities of rats were opened to taking out a blood sample of 5 ml from inferior vena cava for detecting cTnI. Then, randomly selected 15 rats per group were ready for histological handling and other 10 rats were ready for the detection of western blot. In brief, thoracic cavities of these 15 rats were open and injected 1% heparin sodium into the left ventricle from the apex cordis with an infusion needle for anticoagulation, then perfused with 0.85% saline. The inferior vena cava was cut to let the perfusate flow out until it was colorless. Further perfusing 4% paraformaldehyde until the rats stiffened, the heart was removed and put into 4% paraformaldehyde for 24 h fixation, then embedded in paraffin for the standby. The other 10 hearts in each group, which were not perfused, were quickly removed into liquid nitrogen to be stored at − 80 °C.

### Detection of cardiac troponin I in plasma

Automated immunochemiluminescence on an Access 2 immunoassay system (Beckman Coulter, USA) was used to measure cardiac troponin I (cTnI) in plasma with a sensitivity of 0.01 ng/ml. An antibody against human cTnI was used, as the amino acid sequences of human and rat cTnI are 92.8% homologous.

### Chromotrope-2R brilliant green staining

C-2R BG staining is a special method to detect ischemia–hypoxia cardiomyocytes. This method is highly sensitive to ischemia–hypoxia cardiomyocytes, the ischemia–hypoxia cardiomyocytes are stained red, while the normal cardiomyocytes are stained green, and therefore ischemia–hypoxia changes in myocardium can be determined by using C-2R BG staining.

After deparaffinization, the 4-μm-thick slices were stained with hematoxylin and then put into chromotrope-2R for 10 min. The slices were then washed in 0.2% glacial acetic acid three times and incubated in 0.5% brilliant green solution mixed with 70% alcohol for 15 min. Finally, the slices were conventionally dehydrated, made transparent, and sealed with neutral gum. An optical photographic microscope (DP80, Olympus, Tokyo, Japan) was used to obtain the images. Five samples were taken from each group and each sample provided five visual fields, yielding 25 images from each group for statistical analysis. Image-Pro Plus (Media Cybernetics, Silver Spring, MD, USA) was used to measure the integrated optical density (IOD) and the positive ischemia–hypoxia red areas under the same magnification. The mean optical density (MOD, IOD/positive area) was calculated to determine the degree of myocardial ischemia–hypoxia per unit area.

### Immunohistochemical staining

After deparaffinization, the cardiomyocyte slices were washed three times in phosphate buffered saline (PBS) prior to digestion by pepsin complex at room temperature for 8–10 min. Goat serum was used for tissue blocking. The slices were then incubated for 24 h at 4 °C with primary rabbit antibodies against LC3 (anti-rat, 1:200, Novus, CO, USA#NB100-2331-0.1 M), p62 (anti-rat, 1:200, Sigma, CA, USA# SAB3500430-100U), Cathepsin D (anti-rat, 1:200, Santa Cruz, CA, USA #sc-10725), s and Beclin 1 (anti-rat, 1:200, Santa Cruz, CA, USA# sc-11427). To the negative control, such primary antibodies were replaced by antibody diluent. After overnight incubation, the slices were washed in PBS, incubated with a streptavidin–biotin complex kit, and stained brown by diaminobenzidine/peroxidase substrate. The nuclei were stained blue by hematoxylin. An optical photographic microscope (DP80, Olympus, Japan) was used to obtain 25 images from 5 samples in each group, with each sample providing 5 visual fields. Image-Pro Plus (Media Cybernetics, Silver Spring, MD, USA) was used to measure the integrated optical density (IOD), which represented the level of autophagic protein expression, and the positive reaction areas, which represented the areas of autophagic protein expression. From these values, the MOD (IOD/positive area) was calculated, which represented the degree of expression of the autophagic proteins in the cardiomyocytes per unit area.

### Immunohistochemistry and C-2R BG staining in adjacent slices

To investigate the relationship between the expression of autophagic proteins and ischemia–hypoxia in myocardium, both immunohistochemical staining of Beclin1, LC3, Cathepsin D, p62 and C-2R staining were performed in adjacent slices. Rat myocardial tissue with exercise-induced ischemia–hypoxia was used as a control. The negative control of immunohistochemical staining was applied with aforesaid procedure. Images were captured at the same position for each adjacent slice by using microscopic examination (DP80, Olympus, Japan).

### Western blotting

A 30–40 mg section of myocardial tissue was taken from the left ventricle and homogenized to yield the tissue lysate. Thirty micrograms of protein from each sample were separated by sodium dodecyl sulfate-polyacryl-amide gel electrophoresis (SDS-PAGE) and then transferred onto polyvinylidene difluoride (PVDF)-plus membranes at 4 °C. After tissue blocking with 5% bovine serum albumin (BSA), the membranes were incubated overnight at 4 °C with the same four primary rabbit antibodies (1:3000 dilution) used in immunohistochemical staining and GAPDH (FL-335, anti-rat, 1:3000, Santa Cruz, CA, USA). The membranes were then washed three times in tris-buffered saline with 0.1% Tween-20 (TBST) and incubated with HRP-labeled secondary antibody (anti-rabbit IgG, 1:3000; Servicebio Technology, Wuhan, China) at room temperature for 1 h. Finally, the membranes were washed four times in TBST and then imaged using chemiluminescence. Relative densitometry was performed by using a computerized software package (Tanon 5200 Multi Automatic Chemiluminescence Image Analysis system).

### Statistical analysis

All statistical analyses were performed using a statistical software package (SPSS 20.0, Chicago, IL, USA). Data are reported as mean ± SD, and the differences between different groups were compared using one-way ANOVA with the LSD test. *P *< 0.05 indicated statistically significant differences.

## Results

### Exercise preconditioning reduced myocardial ischemia–hypoxia injury from high-intensity exercise

**Fig. 1:**
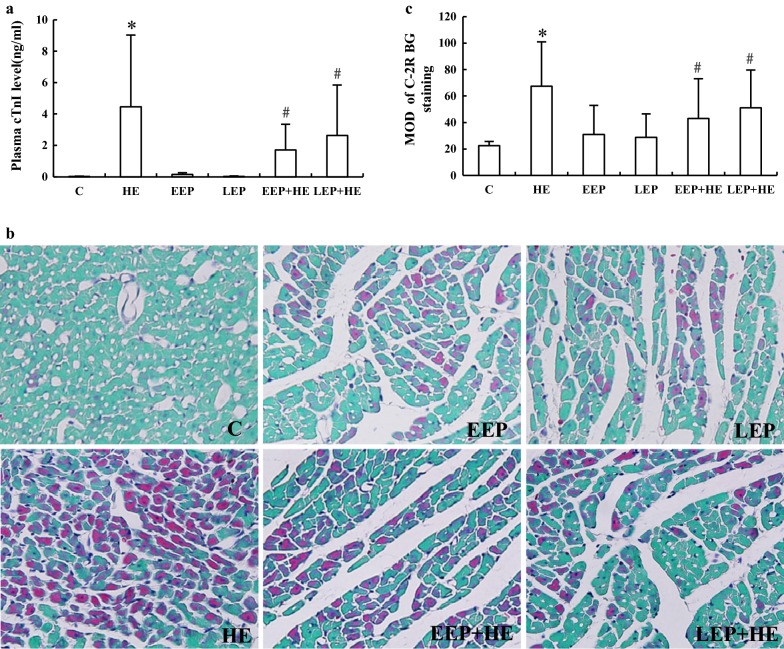
Exercise preconditioning significantly reduces myocardial ischemia–hypoxia injury from high-intensity exercise. **a** Changes in plasma cTnI levels. **b** C-2R BG ischemia–hypoxia staining (×400). The normal cardiomyocytes were green in C-2R BG staining, while the ischemia–hypoxia cardiomyocytes were stained red. **c** Image analysis of C-2R BG ischemia–hypoxia staining. *P < 0.05 vs. group C; ^#^*P *< 0.05 vs. group HE

Changes in plasma cTnI levels reflect the degree of myocardial injury (Fig. 1a). The level of plasma cTnI in the HE group was significantly higher than in the C group (*P *< 0.05), but no significantly different values were observed in the EEP and LEP groups. Plasma cTnI levels in the EEP + HE and the LEP + HE groups were much lower than in the HE group (*P *< 0.05). There was no difference between the EEP + HE and LEP + HE groups.

C-2R BG staining shows the degree of ischemia–hypoxia in the myocardium (Fig. 1b). The ischemia–hypoxia cardiomyocytes were stained red, while the normal cardiomyocytes were stained green. Among all six groups, the nuclei of the cardiomyocytes were light blue and located in the centers. In the C group, the boundaries of all the cardiomyocytes were clear, and they were uniformly stained green (Fig. 1b, c). In the HE group, the boundaries of some of the cardiomyocytes were not clear. Most of the cardiomyocytes were red and only a few were green, indicating that most of the cardiomyocytes were affected by ischemia and hypoxia to different degrees (Fig. 1b-HE). In the EEP and the LEP groups, the boundaries of the cardiomyocytes were clear, with only a few stained red scattered in the tissues (Fig. 1b-EEP, LEP). The number of red-stained cardiomyocytes was much lower in the EEP + HE and the LEP + HE groups than in the HE group (Fig. 1b-HE, EEP + HE, LEP + HE). Analysis of C-2R BG staining (Fig. 1c) showed that the MOD of the HE group was significantly higher than that of the C group (*P *< 0.05), and that there was no difference between either the EEP or the LEP group and the C group. Moreover, the MOD of the EEP + HE and LEP + HE groups were significantly lower than that of the HE group (*P *< 0.05). There were no differences between the EEP + HE and the LEP + HE groups.

These results suggested that EP is a safe way to exercise, as far as the myocardium is concerned, and that during EEP and LEP, EP-induced cardioprotection could reduce the injury from high-intensity exercise.

### Relationship between myocardial ischemia–hypoxia and the expression patterns of autophagy-associated proteins in adjacent slices

**Fig. 2:**
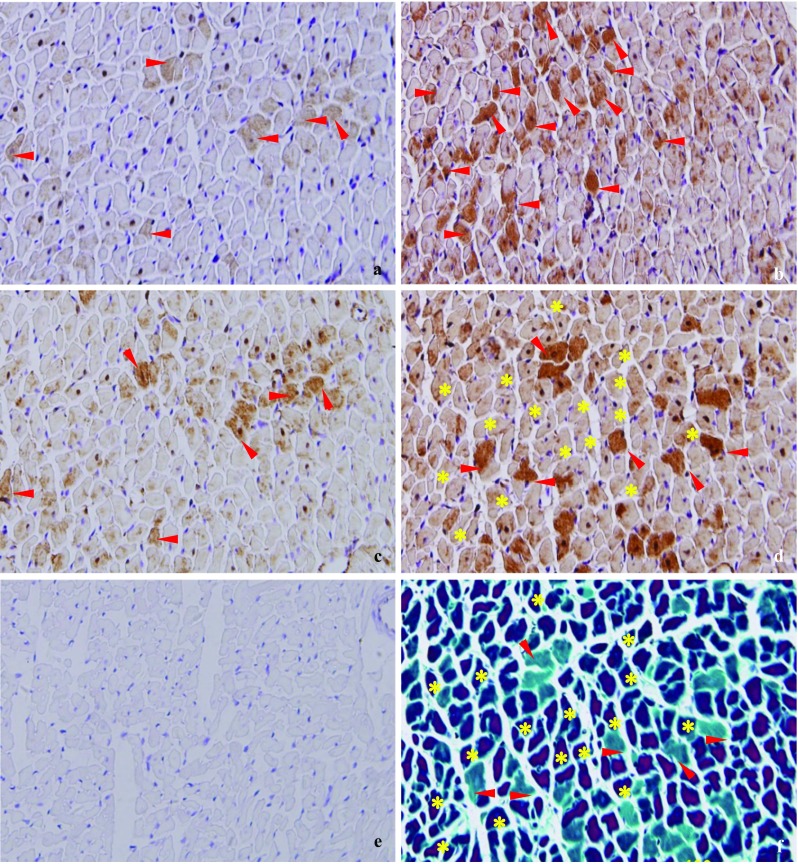
Relationship between myocardial ischemia–hypoxia and autophagy-associated proteins in adjacent slices (×400). The positive immunohistochemical staining of **a** Beclin1, **b** LC3, **c** Cathepsin D and **d** p62 in the cardiomyocytes were stained brown. **e** The negative control of immunohistochemical staining. The ischemia–hypoxia cardiomyocytes were stained red by **f** C-2R BG staining. The normal cardiomyocytes stained green by C-2R BG staining **f** had one-to-one correspondence to p62 positive immunohistochemical cardio myocytes stained brown **d**. Yellow asterisks indicate the areas of ischemia–hypoxia cardiomyocytes stained red by C-2R BG corresponded to the negative p62 staining (light brown). Red arrows indicate the normal cardiomyocytes, stained green by C-2R BG, corresponded completely to positive p62 staining (brown), and incompletely to positive Beclin1 and Cathepsin D. Positive area of LC3 (d, red arrows) had correlation with nonpositive area of p62 (d, yellow asterisks). As shown in red arrows, there is overlap between positive areas of Beclin1 (a) and Cathepsin D (c)

To investigate the relationship between exercise-induced myocardial ischemia–hypoxia and the expression patterns of autophagy-associated proteins, C-2R BG and immunohistochemical staining of the four autophagy-associated proteins (Beclin1, LC3, Cathepsin D, and p62) in adjacent slices were analyzed (Fig. 2). Immunohistochemical staining (brown) revealed that Beclin1 was unevenly distributed in cytoplasm (Fig. 2a), LC3 (Fig. 2b) and Cathepsin D (Fig. 2c) gathered into patches in the cytoplasm, and p62 was spread out in the cytoplasm (Fig. 2d), when they were compared with negative control (Fig. 2e) in which no positive immunohistochemical cardiomyocytes stained brown were found. Interestingly, as is shown by the red arrows in Fig. 2, the normal cardiomyocytes, stained green by C-2R BG (Fig. 2f), corresponded completely to positive p62 staining (brown), and incompletely to positive Beclin1, LC3 and Cathepsin D. The ischemia–hypoxia cardiomyocytes, stained red by C-2R BG (Fig. 2f), were consistent in location with the cardiomyocytes negatively stained by p62 immunohistochemistry (Fig. 2d). Moreover, LC3 (Fig. 2b) had a negative correlation in protein expression with p62 (Fig. 2d), Beclin1 (Fig. 2a) had a positive correlation in protein expression with Cathepsin D (Fig. 2c). These results suggested that myocardial ischemia–hypoxia could induce autophagy.

### Immunohistochemical analysis of the expression of autophagy-associated proteins during exercise preconditioning

**Fig. 3:**
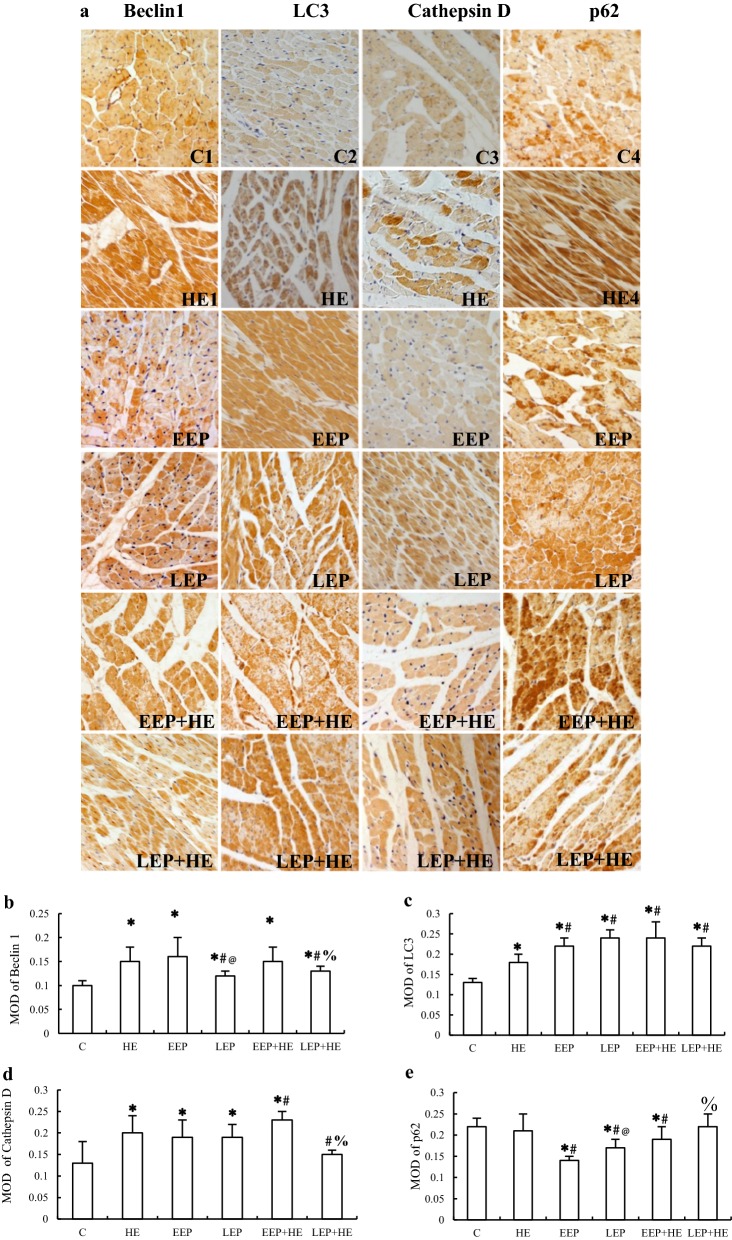
Expression of autophagy-associated proteins in myocardium during EP. **a** Immunohistochemical staining of Beclin1, LC3, Cathepsin D, and p62 (×400). Image analysis of immunohistochemical staining of **b** Beclin1, **c** LC3, **d** Cathepsin d, and **e** p62. *P < 0.05 vs. Group C; ^#^*P *< 0.05 vs. Group HE; @ *P *< 0.05 vs. Group EEP;  %*P *< 0.05 vs. Group EEP + HE

To determine the changes in the expression of autophagy-associated proteins during EP, immunohistochemical staining was performed. Autophagy-associated proteins were stained brown, while the nuclei were stained light blue by hematoxylin. Positively stained cardiomyocytes were clustered into patches in the myocardium (Fig. 3a). Staining revealed that Beclin1 was unevenly distributed throughout the cytoplasm, LC3 was distributed in patches in a few parts of the cytoplasm, and both Cathepsin D and p62 were distributed in patches throughout the cytoplasm. Image analysis showed that the expression of both Beclin1 (Fig. 3b) and LC3 (Fig. 3c) were significantly higher in all of the test groups than in the C group (*P *< 0.05), and expression of Cathepsin D (Fig. 3d) was significantly higher in the HE, EEP, LEP, and EEP + HE groups than in the C group (*P *< 0.05). By contrast, the expression of p62 (Fig. 3e) was significantly lower in the EEP, LEP and EEP + HE groups than in the C group (*P *< 0.05). Image analysis also showed that the expression of Beclin1 was significantly lower in the LEP and LEP + HE groups than in the HE group (*P *< 0.05), and the expression of LC3 was significantly higher in the EEP, LEP, EEP + HE, and LEP + HE groups than in the HE group (*P *< 0.05). Moreover, compared to the HE group, the expression of Cathepsin D was significantly higher in the EEP + HE group (*P *< 0.05) and significantly lower in the LEP + HE group (*P *< 0.05), while the expression of p62 was significantly lower in the EEP, LEP, and EEP + HE than in the HE group (*P *< 0.05). Image analysis also revealed that the expression of both Beclin1 and Cathepsin D was significantly lower and the expression of p62 significantly higher in the LEP + HE group than in the EEP + HE group (*P *< 0.05).

### Western blot analysis of the abundance of autophagy-associated proteins during exercise preconditioning

**Fig. 4:**
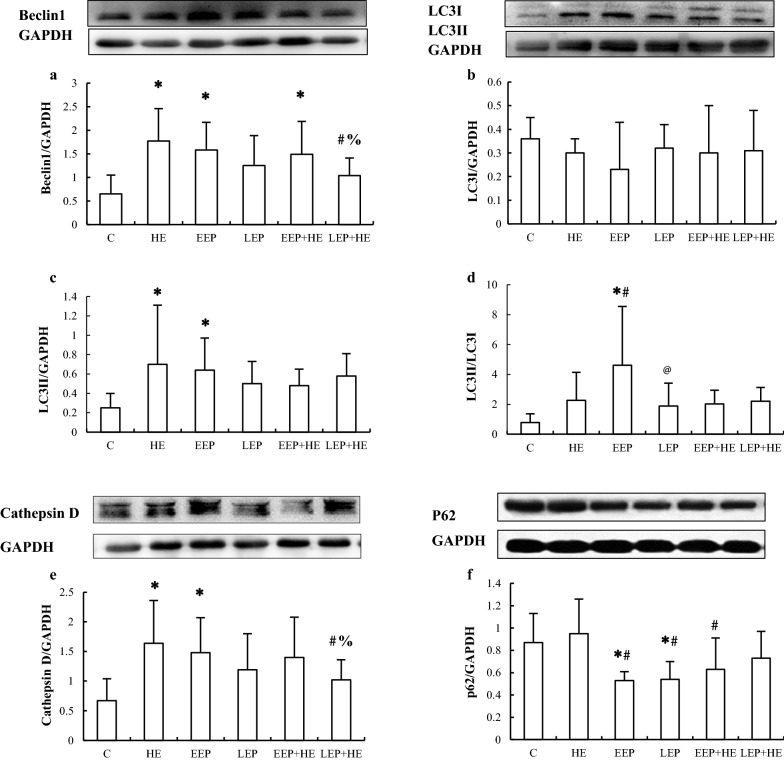
Levels of autophagy-associated proteins in myocardium during EP. **a** Beclin1, **b** LC3-I, **c** LC3-II, **e** Cathepsin D, and **f** p62 were detected by western blotting. **d** Quantitative analysis of LC3-II/LC3-I. *P < 0.05 vs. Group C; ^#^*P *< 0.05 vs. Group HE; @*P *< 0.05 vs. Group EEP;  %*P *< 0.05 vs. Group EEP + HE

The protein abundance of the four autophagy-associated proteins in the myocardium was determined by western blot analysis (Fig. 4). Compared to the C group, Beclin1 (*P *< 0.05, Fig. 4a) levels were significantly higher in the HE, EEP, and EEP + HE groups (*P *< 0.05), LC3II (Fig. 4c) and Cathepsin D (Fig. 4e) levels were significantly higher in the HE and EEP groups (*P *< 0.05), and p62 (Fig. 4f) levels were significantly lower in the EEP and LEP groups (*P *< 0.05). Moreover, the LC3II/LC3I ratio was significantly higher in the EEP group (*P *< 0.05) compared to the C group (Fig. 4d). p62 levels were significantly lower in the EEP and the LEP groups compared to the HE group (*P *< 0.05), while Beclin1 and Cathepsin D levels were significantly lower in the LEP + HE group than in the EEP + HE group (*P *< 0.05). These results suggested that EP could induce autophagy, and that autophagy may play some role in EP-induced cardioprotection.

## Discussion

### Exercise preconditioning reduces high-intensity exercise-induced myocardial ischemia–hypoxia injury

Continuous high-intensity exercise has been associated with myocardial injury, as demonstrated by the increase of certain bio-markers, such as cTnI, in the blood [22]. We have found that cTnI levels were up-regulated by high-intensity exercise, indicating a damage-phenotype. According to our previous reports, cTnI leakage was linked to ultrastructural injuries, such as the breakage of myofibrils, which may be caused by excessive workload and result in mechanical damage to the heart [12, 23]. Furthermore, exercise was found to play a two-faced role in affecting the heart to generate protection or injury [9, 24]. Rats in the EEP and LEP groups were found to not have the significant increase in plasma cTnI levels seen in the HE groups, indicating that the reduction of the time spent continuously running on the treadmill had an effect. The EP and high-intensity exercise used in our study had similar intensities to previous studies, indicating that exercise volume dictates the differences between intermittent EP and sustained high-intensity exercise.

Other than cTnI-driven structural damage, detection of the changes in the level of exercise-induced ischemia–hypoxia is an important tool to systematically assess myocardial injury. We used hematoxylin–basic fuchsin–picric acid (HBFP) staining to detect ischemia–hypoxia based on the principle that there is eosinophilic reinforcement in ischemia–hypoxia region [10, 25]. Similar results were found in the HE group by the C-2R BG staining [26]. Image analyses revealed that both EEP and LEP significantly reduced ischemia–hypoxia due to an adaptive promotion to high-intensity exercise. While mild ischemia–hypoxia changes can be also observed in the EEP and LEP groups, such positive regions fewer and more limited compared to those observed in the HE group. These data suggested a possible association between ischemia–hypoxia and high-intensity exercise. Oxidative stress is a key endogenous mechanism of ischemia–hypoxia [27].

We observed elevated cTnI levels in the EEP + HE and LEP + HE groups but not in the EEP and LEP groups, indicating that the difference was due to the high-intensity exercise. However, we found that EEP and LEP both significantly suppress potential high-intensity exercise-injury in the EEP + HE and LEP + HE groups, confirming the existence of two protective periods [8–10]. Furthermore, cardiac ATP synthesis relies on the supply of oxygen and glucose from coronary circulation. During exercise, sufficient ATP generation through prior adaption conferred by IP and EP allows the heart to avoid negative compensations from heart dysfunction, especially excessive heart rate and decreased ejection fraction [23, 28].

### Exercise preconditioning induces autophagy by intermittent myocardial ischemia–hypoxia

Immunohistochemistry and C-2R BG staining in adjacent slices of myocardium showed that ischemia–hypoxia cardiomyocytes were negatively correlated with the positive immunohistochemical staining of p62. This phenomenon indicated that autophagy could be induced in cardiomyocytes by ischemia and hypoxia. EP typically causes ischemia–hypoxia in a few parts of cardiomyocytes, leading to the induction of autophagy. The intermittent relative or absolute myocardial ischemia and hypoxia caused by repeated short-term, high-intensity intermittent aerobic exercise improves myocardial tolerance to ischemia and hypoxia, thus alleviating the myocardial injuries caused by subsequent long-term ischemia and hypoxia. The expression of ischemia-related autophagy proteins were elevated in the EEP group, with the exception of p62, which was decreased. Our results show that only Beclin1 levels were increased in the LEP group, indicating that EP-intermittent ischemia could induce cellular autophagy for at least a full day. High-intensity exercise was shown to also induce adaptions to ischemia–hypoxia in which autophagy was induced and activated, as indicated by the increased expression of autophagy-associated proteins.

In the energy deficiency heart, activated autophagy plays a key protective role in energy recycling, which may be triggered by EP-induced AMPK signaling [29, 30]. Comparison of ischemia–hypoxia staining and immunohistochemical staining between adjacent myocardial section revealed that cardiomyocytes that did not display signs of ischemia–hypoxia were positively stained for the autophagic substrate p62. Moreover, cardiomyocytes displaying signs of ischemia–hypoxia had significantly different expression levels of autophagy-associated proteins than those that did not—LC3II expression increased, while p62 significantly decreased. These data provide direct evidence of ischemia–hypoxia-induced autophagic flux to the heart undergoing exercise. Ogura, et al. [31] have reported that various autophagic proteins presented different peak points following single-bout exercise. For example, LC3II expression levels were lowest immediately after exercise, rose to a peak 1 h later, and then surpassed the levels measured prior to exercise. However, in that report, p62 levels never changed. Combined with our observations, this indicates that it is difficult to acquire a pair with analogous expression levels of autophagy proteins.

We speculated that the aforementioned phenomenon could be explained by autophagic flux. In cardiomyocytes, Beclin1 expression may positively correspond to Cathepsin D expression, but LC3 expression was potentially negatively correlated to p62 expression. It has been reported that increased LC3 and decreased p62 together indicate autophagic activation [6]. Furthermore, Beclin1 and Cathepsin can both be induced by ischemia, which is essential to IP-induced cardioprotection [32]. While exercise has been previously reported to induce autophagy via hypoxia [33], our results showed that various autophagy-associated proteins were activated during different stages of autophagy in individual cardiomyocytes. This suggests that different autophagic processions may be important in the prevention of extensive damage in ischemia–hypoxia myocardium [43], where increases in p62 levels in the non-ischemia–hypoxia region can predict the subsequent consumption of autophagic substrates [34].

Our data suggest a connection between Beclin1 and Cathepsin D, both of which have similar expression level trends among the experimental groups. In the HE group, Beclin1, LC3, and Cathepsin D were up-regulated, while p62 expression was unchanged. This indicated that high-intensity exercise was associated with apparent autophagic induction, which was accompanied by high levels of ischemia–hypoxia. However, induction of autophagy in this situation may be impeded due to the reduced availability of the key substrate, p62. Autophagic proteins may fail to be properly translocated during excessive exercise, e.g., damaged mitochondria and misfolded proteins, which leads to the reduction of autophagic efficiency [6, 12]. Yan, et al. [35] have reported that maintaining a normal level of autophagy during exercise is essential to cardiac metabolism and cardioprotection. Our results show that the EEP group has high-intensity exercise-like autophagic induction, despite the decreased expression of p62 and the increased ratio of LC3II/LC3I. EEP-induced intermittent ischemia–hypoxia played a more significant role in the induction of autophagy than did high-intensity exercise. Based on these data, we speculated that the different autophagy phenotypes observed in high-intensity exercise and EEP were due to the different degrees to which they induced ischemia–hypoxia. Autophagy in the LEP group was nearly recovered to the levels before EP, demonstrating that EP-induced autophagic flux concludes within 24 h. Ma et al. [36] have reported that LC3 content and mRNA expression gradually increased within 12 h after myocardial infarction, but the peak of many autophagy inducers at 6 h then fell back within next 6 h. Likewise, total LC3, Cathepsin D, and p62 levels in the LEP groups presumably stay at their basal levels since they were altered by EEP, which may play a positive role in their protection.

### Exercise preconditioning-regulated autophagy assists cardioprotection

Relative to the HE group, almost all autophagic markers in the EEP + HE and LEP + HE groups were at a normal level, demonstrating that autophagic flux was unobstructed. This suggests that autophagic flux might provide the additional energy required by the myocardium during ischemia–hypoxia. During subsequent high-intensity exercise, autophagy was involved in the cardioprotection of EP and alleviated cTnI leakage in cardiomyocytes, attenuating the myocardial ischemia–hypoxia caused by high-intensity exercise.

Taken together, these results suggest that high-intensity exercise-induced myocardial injury and ischemia–hypoxia can be inhibited by EP during both early and late windows. In both EP and IP, the autophagy maintenance of ATP synthesis capacity is crucial to generating the adaptive promotion for additional cardiovascular stress. Such effects could be eliminated by PI3K inhibition, which is a possible mechanism to explain the suppression of ischemia–hypoxia in high-intensity exercise [12, 37]. However, disruption between the autophagosome and lysosomes may reduce the efficiency of autophagy, resulting in interruption of the autophagic flux [38]. We found that the levels of Beclin1 and Cathepsin D in the EEP + HE group were as high as those in the EEP group, indicating that they were not affected by high-intensity exercise. However, the LC3II levels and the ratio of LC3II/LC3I decreased and the p62 levels increased in the EEP + HE group compared to the EEP group. Through a modified ubiquitin system, pro-LC3 in EEP may be transformed to LC3I and then to LC3II, and this process plays key role in autophagic phospholipid membrane [39]. The fusion between the outer-membrane of the autophagosome and the mono-membrane of the lysosome generates a new structure, the autolysosome. In this case, proteins at the inner-membrane of the autophagosome, such as LC3II and p62 in EEP + HE, and encapsulated substrates are hydrolyzed by proteases, e.g., EEP-assisted Cathepsin D in the lysosome. As such, p62 is considered a marker of activated autophagy in EEP-cardioprotection [40]. Multiple lysosomal enzymes contribute to protein degradation and to the cellular recycling of amino acids [41]. During IP, LC3II-marked induction of autophagy is accompanied by increases in the interaction between Beclin1 and Bcl-2. This interaction is key, as IP-protection inhibits reperfusion-induced Beclin1 overexpression and increases in p62 consumption [42]. Differences in the expression of autophagy-associated proteins have been observed between repetitive coronary occlusion and repetitive stenosis, the latter of which has a lower infarct size and a higher expression of Beclin1, LC3II/I, and Cathepsin B than subsequent continuous occlusion [43]. In these cases, the autophagic protection induced by EEP + HE is associated with an improved cellular environment, potentially better than that induced by IP intervention. However, autophagy levels during high-intensity exercise were suppressed by EEP, indicating that lower levels of autophagy are already sufficient to decrease injury of high-intensity exercise, where adjusted autophagy may be important for the early cardioprotection by EP.

Similar repressive trends were also observed in the LEP + HE group, in which both Beclin1 and Cathepsin D stayed at base-levels during LEP and were not influenced by subsequent high-intensity exercise. These results indicated that EP improved adaptation to high-intensity exercise-induced autophagy via suppressing the expression of autophagic inducers proteins involved in lysosomal function. Therefore, while EP-cardioprotection may involve unobstructed autophagy levels, high-intensity exercise does not. However, autophagy in LEP-protection was likely weaker than that in EEP-protection due to lower expressions of Beclin1, LC3, and Cathepsin D and higher expression of p62. p62 is required for multiple types of selective binding in its expression that enhances the protective effect of both EP and IP [44]. We speculated that other factors, e.g., mitophagy, play more important roles in LEP-protection [12]. The increase in the induction of autophagy during EEP-protection due to prior EEP-induction has more cellular benefits than those conferred by LEP-protection.

## Conclusions

**Fig. 5:**
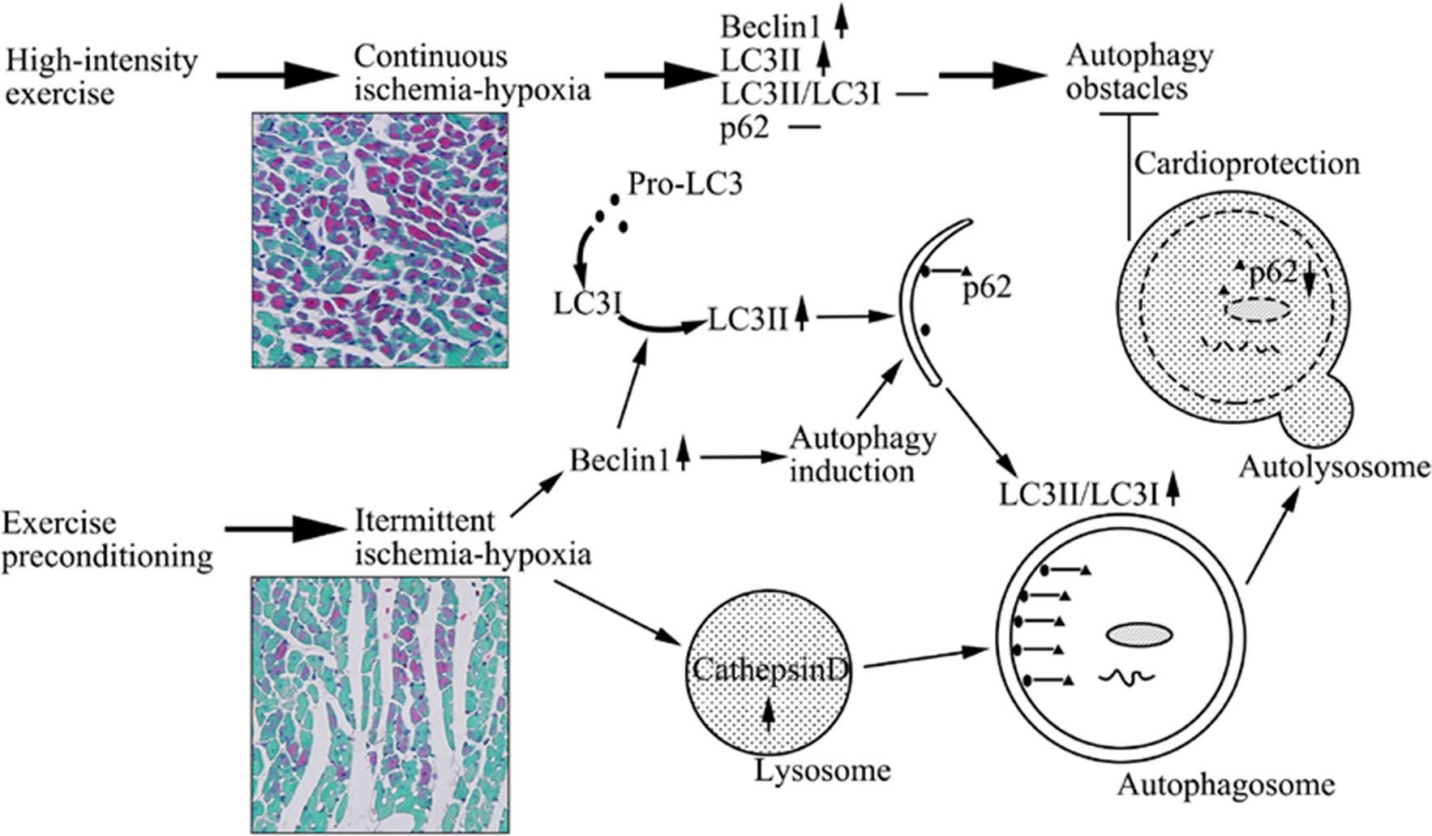
Mechanisms in EP-induced autophagy assisting cardioprotection by intermittent ischemia–hypoxia. Autophagy is initiated by intermittent ischemia–hypoxia of EP by inducing upregulation of Beclin1 levels. The process of autophagy was complete because of the degradation of autophagy substrate p62. High-intensity exercise can also induce autophagy by continuous ischemia–hypoxia. However, it fails to increase LC3II/LC3I ratio, and to decrease p62, which relate to autophagy obstacles. Preraised autophagy by EP-induced ischemia–hypoxia plays an adaptive role in subsequent acute stress, allowing heart to be protected

EP significantly suppresses high-intensity exercise-induced myocardial injury and ischemia–hypoxia in both early and late cardioprotection. There is a clear correlation between autophagy and exercise-induced ischemia–hypoxia. High-intensity exercise-induced continuous ischemia–hypoxia and EP-induced intermittent ischemia–hypoxia both result in the induction of autophagy, although high-intensity exercise-induced autophagy was less efficient. While levels of autophagy decreased in a time-dependent manner from EEP to LEP, during both cardioprotective windows, increased autophagy by EEP had adaptive effects. Through the suppression of the expression of proteins potentially involved in the obstruction of high-intensity exercise-induced autophagy, autophagy was maintained at a high efficiency, assisting the generation of cardioprotection presumably due to adaptation via intermittent ischemia–hypoxia (Fig. 5).

## Data Availability

The data used to support the findings of this study are available from the corresponding author upon request

## Abbreviations

EP: Exercise preconditioning
IP: Ischemic preconditioning
LC3: Microtubule-associated protein 1A/1B light chain 3
p62: SQSTM1/sequestosome 1
C-2R BG: Chromotrope-2R brilliant green staining
IOD: Integrated optical density
MOD: Mean optical density
GADPH: Glyceraldehyde-3-phosphate dehydrogenase
AMPK: AMP-activated protein kinase
